# Supplementary material for: Magnitude and nucleation time of the 2017 Pohang Earthquake point to its predictable artificial triggering
Source: Nat Commun. 2021 Nov 4;12:6397. doi: 10.1038/s41467-021-26679-w (PMC8568929; doi:10.1038/s41467-021-26679-w)
Supplement: Supplementary file 1 — Supplementary Information [file 41467_2021_26679_MOESM1_ESM.pdf]

> Magnitude and nucleation time of the 2017 Pohang Earthquake point to its predictable artificial triggering.

Serge A. Shapiro, Kwang-Hee Kim, Jin-Han Ree

Supplementary Material

#A MapleWorksheet for computation of Figure 1.

DT, DTFld=Elapsed Time Vectors; MAG, MwFld=Magnitude Vectors; MOM=Moments; VOL,DVFld=Injected Volumes until event occurrence;

> *DT* := *Vector*[*row*](*readdata*(*time_to_event*, *float*)); *DTFld* := *Vector*[*row*](*readdata*(*DtField*, *float*)); #*All Scales and Field Scale only data*

$DT := [65000., 29000., 430000., 1.2 \times 10^6, 2.1 \times 10^7, 520000., 3.2 \times 10^7, 860000., 4.6 \times 10^8, 3.2 \times 10^7, 1.2 \times 10^8, 4.1 \times 10^8, 2.0 \times 10^7, 3.5 \times 10^8, 1.7 \times 10^8, 1.7 \times 10^8, 4.9 \times 10^8, 5.7 \times 10^8, 5.8 \times 10^8, 5.7 \times 10^8, 6.0 \times 10^8, 1.7 \times 10^8, 1.7 \times 10^6, 63000., 240000., 200000., 43000., 7200., 5400., 12000., 13000., 140000., 95000., 17000., 130000., 220000., 5.2 \times 10^6, 16000., 12000., 7200., 3000., 3000., 2400., 257.0, 552.0, 520.0, 130.0, 60.0, 8400.0, 3600.0, 14400.0, 12600.0, 5400.0, 17400.0, 6.35 \times 10^7]$

$DTFld := [65000., 29000., 430000., 1.2 \times 10^6, 2.1 \times 10^7, 520000., 3.2 \times 10^7, 860000., 4.6 \times 10^8, 3.2 \times 10^7, 1.2 \times 10^8, 4.1 \times 10^8, 2.0 \times 10^7, 3.5 \times 10^8, 1.7 \times 10^8, 1.7 \times 10^8, 4.9 \times 10^8, 5.7 \times 10^8, 5.8 \times 10^8, 5.7 \times 10^8, 6.0 \times 10^8, 1.7 \times 10^8, 1.7 \times 10^6, 63000., 240000., 200000., 43000., 7200., 5400., 12000., 13000., 140000., 95000., 17000., 130000., 220000., 5.2 \times 10^6, 16000., 12000., 7200., 3000., 3000., 2400., 6.35 \times 10^7]$ **(1)**

> *MAG* := *Vector*[*row*](*readdata*(*magnitude*, *float*)); *MwFld* := *Vector*[*row*](*readdata*(*MwField*, *float*)); #*All Scales and Field Scale only data*

$MAG := [1.4, 2.3, 3.0, 2.9, 3.3, 3.4, 3.6, 3.7, 3.9, 4.0, 4.3, 4.4, 4.7, 4.8, 4.85, 4.8, 5.3, 5.7, 5.8, 5.1, 5.0, 4.0, 3.4, 3.0, 2.5, 2.4, 2.4, -1.2, -0.6, -1.7, -2.0, 1., 0.1, 0.3, 2.4, 1.3, 2.0, 0.1, 0., -0.3, -1.0, -0.8, -0.4, -7.0, -7.45, -7.43, -6.84, -7.24, -3.2, -3.63, -3.5, -3.4, -3.9, -3.14, 5.5]$

$MwFld := [1.4, 2.3, 3.0, 2.9, 3.3, 3.4, 3.6, 3.7, 3.9, 4.0, 4.3, 4.4, 4.7, 4.8, 4.85, 4.8, 5.3, 5.7, 5.8, 5.1, 5.0, 4.0, 3.4, 3.0, 2.5, 2.4, 2.4, -1.2, -0.6, -1.7, -2.0, 1., 0.1, 0.3, 2.4, 1.3, 2.0, 0.1, 0., -0.3, -1.0, -0.8, -0.4, 5.5]$ **(2)**

> *MOM* := *Vector*[*row*](*readdata*(*moment*, *float*)); #*All Scales data*

$MOM := [1.43 \times 10^{11}, 3.2 \times 10^{12}, 3.5 \times 10^{13}, 2.51 \times 10^{13}, 8.9 \times 10^{13}, 1.41 \times 10^{14}, 2.82 \times 10^{14}, 3.98 \times 10^{14}, 8.0 \times 10^{14}, 8.3 \times 10^{14}, 3.16 \times 10^{15}, 4.5 \times 10^{15}, 1.2 \times 10^{16}, 2.0 \times 10^{16}, 2.1 \times 10^{16}, 2.21 \times 10^{16}, 1.0 \times 10^{17}, 3.92 \times 10^{17}, 4.16 \times 10^{17}, 5.6 \times 10^{16}, 3.3 \times 10^{16}, 1.0 \times 10^{15}, 1.41 \times 10^{14}, 3.5 \times 10^{13}, 6.3 \times 10^{12}, 5.0 \times 10^{12}, 5.0 \times 10^{12}, 2.0 \times 10^7,$ **(3)**

1.6×10^{8}, 3.6×10^{6}, 1.3×10^{6}, 3.98×10^{11}, 1.78×10^{9}, 3.55×10^{9}, 1.0×10^{13}, 1.1×10^{11}, 1.26×10^{12}, 1.78×10^{9}, 1.26×10^{9}, 4.47×10^{9}, 3.98×10^{7}, 7.94×10^{7}, 3.16×10^{8}, 0.04, 0.01, 0.01, 0.06, 0.01, 19952.6, 4518.6, 7079.5, 24547.1, 1778.3, 24547.1, 2.34×10^{17}]

```
> VOL := Vector[row](readdata(volume_to_event, float)); DVFld := Vector[row](readdata(DVField, float)); #All Scales and Field Scale only data
```

VOL := [200., 4170., 17500., 39800., 282000., 11500., 61700., 20000., 340000., 83400., 2.7×10^{6}, 426000., 629000., 1.19×10^{6}, 625000., 991000., 7.84×10^{6}, 6.70×10^{6}, 8.84×10^{6}, 3.55×10^{6}, 6.82×10^{6}, 1.08×10^{6}, 19400., 4300., 3100., 350., 1600., 1100., 1200., 400., 1890., 25700., 28800., 13500., 22400., 21600., 70000., 3100., 2200., 2700., 300., 300., 192., 4.3×10^{-6}, 2.3×10^{-6}, 4.4×10^{-6}, 4.4×10^{-6}, 4.1×10^{-6}, 1.78, 0.38, 1.03, 0.66, 0.12, 2.01, 5840.]

DVFld := [200., 4170., 17500., 39800., 282000., 11500., 61700., 20000., 340000., 83400., 2.7×10^{6}, 426000., 629000., 1.19×10^{6}, 625000., 991000., 7.84×10^{6}, 6.70×10^{6}, 8.84×10^{6}, 3.55×10^{6}, 6.82×10^{6}, 1.08×10^{6}, 19400., 4300., 3100., 350., 1600., 1100., 1200., 400., 1890., 25700., 28800., 13500., 22400., 21600., 70000., 3100., 2200., 2700., 300., 300., 192., 5840.] **(4)**

```
> T3N1DMo := 3·log10~(DT); LDTField := log10~(DTFld); LDVField := log10~(DVFld); #LogTimes, LogVolumes
```

T3N1DMo := [14.4387400710000, 13.3871939940000, 16.9004053680000, 18.2375437380000, 21.9666578850000, 17.1480100320000, 22.5154499340000, 17.8034953530000, 25.9882734960000, 22.5154499340000, 24.2375437380000, 25.8383515710000, 21.9030899880000, 25.6322041320000, 24.6913467630000, 24.6913467630000, 26.0705882400000, 26.2676245680000, 26.2902839820000, 26.2676245680000, 26.3344537500000, 24.6913467630000, 18.6913467630000, 14.3980216470000, 16.1406337260000, 15.9030899880000, 13.9004053680000, 11.5719974880000, 11.1971812800000, 12.2375437380000, 12.3418300560000, 15.4383841080000, 14.9331708150000, 12.6913467630000, 15.3418300560000, 16.0272680430000, 20.1480100320000, 12.6123599490000, 12.2375437380000, 11.5719974880000, 10.4313637650000, 10.4313637650000, 10.1406337260000, 7.22979936900000, 8.22581723400000, 8.14801003200000, 6.34183005600000, 5.33445375000000, 11.7728378580000, 10.6689075030000, 12.4750874760000, 12.3011116350000, 11.1971812800000, 12.7216477440000, 23.4083211750000]

LDTField := [4.812913357, 4.462397998, 5.633468456, 6.079181246, 7.322219295, 5.716003344, 7.505149978, 5.934498451, 8.662757832, 7.505149978, 8.079181246, 8.612783857, 7.301029996, 8.544068044, 8.230448921, 8.230448921, 8.690196080,

8.755874856, 8.763427994, 8.755874856, 8.778151250, 8.230448921, 6.230448921, 4.799340549, 5.380211242, 5.301029996, 4.633468456, 3.857332496, 3.732393760, 4.079181246, 4.113943352, 5.146128036, 4.977723605, 4.230448921, 5.113943352, 5.342422681, 6.716003344, 4.204119983, 4.079181246, 3.857332496, 3.477121255, 3.477121255, 3.380211242, 7.802773725]

LDVField := [2.301029996, 3.620136055, 4.243038049, 4.599883072, 5.450249108, 4.060697840, 4.790285164, 4.301029996, 5.531478917, 4.921166051, 6.431363764, 5.629409599, 5.798650645, 6.075546961, 5.795880017, 5.996073654, 6.894316063, 6.826074803, 6.946452265, 6.550228353, 6.833784375, 6.033423756, 4.287801730, 3.633468456, 3.491361694, 2.544068044, 3.204119983, 3.041392685, 3.079181246, 2.602059991, 3.276461804, 4.409933123, 4.459392488, 4.130333768, 4.350248018, 4.334453751, 4.845098040, 3.491361694, 3.342422681, 3.431363764, 2.477121255, 2.477121255, 2.283301229, 3.766412847] **(5)**

> *#LogMoments, LogTimes, and Various Non-Linear (N) and Linear (L) Scalings for Moments (Mo) and Magnitudes (Mg) in 1D and 3D, 3r2 and 2r3=3:2,2:3;*

> *#constants added for ploting convenience; they are of no significance; scale exponents (straight line coefficients) are of importance only*

> $MOML := \text{log10}\sim(MOM);\ TL := \text{log10}\sim(DT);\ T3r2LDMo := \left(3.7 + \frac{3}{2}\cdot \text{log10}\right)\sim(DT);$ $T2N1DMg := (-10 + 2\cdot \text{log10})\sim(DT);\ T2r3N3DMg := \frac{2}{3}\cdot \text{log10}\sim(DT);\ T1LDMo :=$ $(8.5 + \text{log10})\sim(DT);\ T3N1DMo := (-3.5 + 3\cdot \text{log10})\sim(DT);\ T1LDMg := \text{log10}$ $\sim(DT);$

MOML := [11.15533604, 12.50514998, 13.54406804, 13.39967372, 13.94939001, 14.14921911, 14.45024911, 14.59988307, 14.90308999, 14.91907809, 15.49968708, 15.65321251, 16.07918125, 16.30103000, 16.32221929, 16.34439227, 17.00000000, 17.59328607, 17.61909333, 16.74818803, 16.51851394, 15.00000000, 14.14921911, 13.54406804, 12.79934055, 12.69897000, 12.69897000, 7.301029996, 8.204119983, 6.556302501, 6.113943352, 11.59988307, 9.250420002, 9.550228353, 13.00000000, 11.04139269, 12.10037055, 9.250420002, 9.100370545, 9.650307523, 7.599883072, 7.899820502, 8.499687083, −1.397940009, −2.000000000, −2.000000000, −1.221848750, −2.000000000, 4.299999496, 3.655003898, 3.850002586, 4.390000192, 3.250005028, 4.390000192, 17.36921586]

TL := [4.812913357, 4.462397998, 5.633468456, 6.079181246, 7.322219295, 5.716003344, 7.505149978, 5.934498451, 8.662757832, 7.505149978, 8.079181246, 8.612783857, 7.301029996, 8.544068044, 8.230448921, 8.230448921, 8.690196080, 8.755874856, 8.763427994, 8.755874856, 8.778151250, 8.230448921, 6.230448921, 4.799340549, 5.380211242, 5.301029996, 4.633468456, 3.857332496, 3.732393760, 4.079181246, 4.113943352, 5.146128036, 4.977723605, 4.230448921, 5.113943352, 5.342422681,

6.716003344, 4.204119983, 4.079181246, 3.857332496, 3.477121255, 3.477121255, 3.380211242, 2.409933123, 2.741939078, 2.716003344, 2.113943352, 1.778151250, 3.924279286, 3.556302501, 4.158362492, 4.100370545, 3.732393760, 4.240549248, 7.802773725]

T3r2LDMo ≔ [10.91937004, 10.39359700, 12.15020268, 12.81877187, 14.68332894, 12.27400502, 14.95772497, 12.60174768, 16.69413675, 14.95772497, 15.81877187, 16.61917579, 14.65154499, 16.51610207, 16.04567338, 16.04567338, 16.73529412, 16.83381228, 16.84514199, 16.83381228, 16.86722688, 16.04567338, 13.04567338, 10.89901082, 11.77031686, 11.65154499, 10.65020268, 9.485998744, 9.298590640, 9.818771869, 9.870915028, 11.41919205, 11.16658541, 10.04567338, 11.37091503, 11.71363402, 13.77400502, 10.00617997, 9.818771869, 9.485998744, 8.915681882, 8.915681882, 8.770316863, 7.314899684, 7.812908617, 7.774005016, 6.870915028, 6.367226875, 9.586418929, 9.034453752, 9.937543738, 9.850555818, 9.298590640, 10.06082387, 15.40416059]

T2N1DMg ≔ [−0.374173286, −1.075204004, 1.26693691, 2.15836249, 4.64443859, 1.43200669, 5.01029996, 1.86899690, 7.32551566, 5.01029996, 6.15836249, 7.22556771, 4.60205999, 7.08813609, 6.46089784, 6.46089784, 7.38039216, 7.51174971, 7.52685599, 7.51174971, 7.55630250, 6.46089784, 2.46089784, −0.401318902, 0.76042248, 0.60205999, −0.733063088, −2.285335008, −2.535212480, −1.841637508, −1.772113296, 0.29225607, −0.044552790, −1.539102158, 0.22788670, 0.68484536, 3.43200669, −1.591760034, −1.841637508, −2.285335008, −3.045757490, −3.045757490, −3.239577516, −5.180133754, −4.516121844, −4.567993312, −5.772113296, −6.443697500, −2.151441428, −2.887394998, −1.683275016, −1.799258910, −2.535212480, −1.518901504, 5.60554745]

T2r3N3DMg ≔ [3.20860890466667, 2.97493199866667, 3.75564563733333, 4.05278749733333, 4.88147953000000, 3.81066889600000, 5.00343331866667, 3.95633230066667, 5.77517188800000, 5.00343331866667, 5.38612083066667, 5.74185590466667, 4.86735333066667, 5.69604536266667, 5.48696594733333, 5.48696594733333, 5.79346405333333, 5.83724990400000, 5.84228532933333, 5.83724990400000, 5.85210083333333, 5.48696594733333, 4.15363261400000, 3.19956036600000, 3.58680749466667, 3.53401999733333, 3.08897897066667, 2.57155499733333, 2.48826250666667, 2.71945416400000, 2.74262890133333, 3.43075202400000, 3.31848240333333, 2.82029928066667, 3.40929556800000, 3.56161512066667, 4.47733556266667, 2.80274665533333, 2.71945416400000, 2.57155499733333, 2.31808083666667, 2.31808083666667, 2.25347416133333, 1.60662208200000, 1.82795938533333, 1.81066889600000, 1.40929556800000, 1.18543416666667, 2.61618619066667, 2.37086833400000, 2.77224166133333, 2.73358036333333, 2.48826250666667, 2.82703283200000, 5.20184915000000]

$T1LDMo := [13.31291336, 12.96239800, 14.13346846, 14.57918125, 15.82221930, 14.21600334, 16.00514998, 14.43449845, 17.16275783, 16.00514998, 16.57918125, 17.11278386, 15.80103000, 17.04406804, 16.73044892, 16.73044892, 17.19019608, 17.25587486, 17.26342799, 17.25587486, 17.27815125, 16.73044892, 14.73044892, 13.29934055, 13.88021124, 13.80103000, 13.13346846, 12.35733250, 12.23239376, 12.57918125, 12.61394335, 13.64612804, 13.47772360, 12.73044892, 13.61394335, 13.84242268, 15.21600334, 12.70411998, 12.57918125, 12.35733250, 11.97712126, 11.97712126, 11.88021124, 10.90993312, 11.24193908, 11.21600334, 10.61394335, 10.27815125, 12.42427929, 12.05630250, 12.65836249, 12.60037054, 12.23239376, 12.74054925, 16.30277372]$

$T3N1DMo := [10.93874007, 9.88719399, 13.40040537, 14.73754374, 18.46665788, 13.64801003, 19.01544993, 14.30349535, 22.48827350, 19.01544993, 20.73754374, 22.33835157, 18.40308999, 22.13220413, 21.19134676, 21.19134676, 22.57058824, 22.76762457, 22.79028398, 22.76762457, 22.83445375, 21.19134676, 15.19134676, 10.89802165, 12.64063373, 12.40308999, 10.40040537, 8.07199749, 7.69718128, 8.73754374, 8.84183006, 11.93838411, 11.43317082, 9.19134676, 11.84183006, 12.52726804, 16.64801003, 9.11235995, 8.73754374, 8.07199749, 6.93136376, 6.93136376, 6.64063373, 3.729799369, 4.725817234, 4.648010032, 2.841830056, 1.834453750, 8.27283786, 7.16890750, 8.97508748, 8.80111164, 7.69718128, 9.22164774, 19.90832118]$

$T1LDMg := [4.812913357, 4.462397998, 5.633468456, 6.079181246, 7.322219295, 5.716003344, 7.505149978, 5.934498451, 8.662757832, 7.505149978, 8.079181246, 8.612783857, 7.301029996, 8.544068044, 8.230448921, 8.230448921, 8.690196080, 8.755874856, 8.763427994, 8.755874856, 8.778151250, 8.230448921, 6.230448921, 4.799340549, 5.380211242, 5.301029996, 4.633468456, 3.857332496, 3.732393760, 4.079181246, 4.113943352, 5.146128036, 4.977723605, 4.230448921, 5.113943352, 5.342422681, 6.716003344, 4.204119983, 4.079181246, 3.857332496, 3.477121255, 3.477121255, 3.380211242, 2.409933123, 2.741939078, 2.716003344, 2.113943352, 1.778151250, 3.924279286, 3.556302501, 4.158362492, 4.100370545, 3.732393760, 4.240549248, 7.802773725]$ **(6)**

> $\frac{20}{1.5} - 6.07; \frac{-2}{1.5} - 6.07;$ *# Mw range of Mmax data plot: -7.403 to 7.263;*

$$7.26333333$$
$$-7.403333333$$ **(7)**

> $VOLL := \log10\sim(VOL); VL3r2p0 := \frac{3}{2}\cdot\log10\sim(VOL); VL3N1DMo := 3\cdot\log10\sim(VOL);$

$VL1N3DMo := 10 +\sim VOLL; VL3r2LDMo := 6 +\sim VL3r2p0;$

VOLL := [2.301029996, 3.620136055, 4.243038049, 4.599883072, 5.450249108, 4.060697840, 4.790285164, 4.301029996, 5.531478917, 4.921166051, 6.431363764, 5.629409599, 5.798650645, 6.075546961, 5.795880017, 5.996073654, 6.894316063, 6.826074803, 6.946452265, 6.550228353, 6.833784375, 6.033423756, 4.287801730, 3.633468456, 3.491361694, 2.544068044, 3.204119983, 3.041392685, 3.079181246, 2.602059991, 3.276461804, 4.409933123, 4.459392488, 4.130333768, 4.350248018, 4.334453751, 4.845098040, 3.491361694, 3.342422681, 3.431363764, 2.477121255, 2.477121255, 2.283301229, −5.366531544, −5.638272164, −5.356547324, −5.356547324, −5.387216143, 0.2504200023, −0.4202164034, 0.01283722471, −0.1804560645, −0.9208187540, 0.3031960574, 3.766412847]

VL3r2p0 := [3.45154499400000, 5.43020408250000, 6.36455707350000, 6.89982460800000, 8.17537366200000, 6.09104676000000, 7.18542774600000, 6.45154499400000, 8.29721837550000, 7.38174907650000, 9.64704564600000, 8.44411439850000, 8.69797596750000, 9.11332044150000, 8.69382002550000, 8.99411048100000, 10.3414740945000, 10.2391122045000, 10.4196783975000, 9.82534252950000, 10.2506765625000, 9.05013563400000, 6.43170259500000, 5.45020268400000, 5.23704254100000, 3.81610206600000, 4.80617997450000, 4.56208902750000, 4.61877186900000, 3.90308998650000, 4.91469270600000, 6.61489968450000, 6.68908873200000, 6.19550065200000, 6.52537202700000, 6.50168062650000, 7.26764706000000, 5.23704254100000, 5.01363402150000, 5.14704564600000, 3.71568188250000, 3.71568188250000, 3.42495184350000, −8.04979731600000, −8.45740824600000, −8.03482098600000, −8.03482098600000, −8.08082421450000, 0.375630003450000, −0.630324605100000, 0.0192558370650000, −0.270684096750000, −1.38122813100000, 0.454794086100000, 5.64961927050000]

VL3N1DMo := [6.90308998800000, 10.8604081650000, 12.7291141470000, 13.7996492160000, 16.3507473240000, 12.1820935200000, 14.3708554920000, 12.9030899880000, 16.5944367510000, 14.7634981530000, 19.2940912920000, 16.8882287970000, 17.3959519350000, 18.2266408830000, 17.3876400510000, 17.9882209620000, 20.6829481890000, 20.4782244090000, 20.8393567950000, 19.6506850590000, 20.5013531250000, 18.1002712680000, 12.8634051900000, 10.9004053680000, 10.4740850820000, 7.63220413200000, 9.61235994900000, 9.12417805500000, 9.23754373800000, 7.80617997300000, 9.82938541200000, 13.2297993690000, 13.3781774640000, 12.3910013040000, 13.0507440540000, 13.0033612530000, 14.5352941200000, 10.4740850820000, 10.0272680430000, 10.2940912920000, 7.43136376500000, 7.43136376500000, 6.84990368700000, −16.0995946320000, −16.9148164920000, −16.0696419720000, −16.0696419720000, −16.1616484290000, 0.751260006900000, −1.26064921020000, 0.0385116741300000,

−0.541368193500000, −2.76245626200000, 0.909588172200000, 11.2992385410000]

VL1N3DMo := [12.30103000, 13.62013606, 14.24303805, 14.59988307, 15.45024911, 14.06069784, 14.79028516, 14.30103000, 15.53147892, 14.92116605, 16.43136376, 15.62940960, 15.79865064, 16.07554696, 15.79588002, 15.99607365, 16.89431606, 16.82607480, 16.94645226, 16.55022835, 16.83378438, 16.03342376, 14.28780173, 13.63346846, 13.49136169, 12.54406804, 13.20411998, 13.04139268, 13.07918125, 12.60205999, 13.27646180, 14.40993312, 14.45939249, 14.13033377, 14.35024802, 14.33445375, 14.84509804, 13.49136169, 13.34242268, 13.43136376, 12.47712126, 12.47712126, 12.28330123, 4.633468456, 4.361727836, 4.643452676, 4.643452676, 4.612783857, 10.25042000, 9.579783597, 10.01283722, 9.819543936, 9.079181246, 10.30319606, 13.76641285]

VL3r2LDMo := [9.45154499400000, 11.4302040825000, 12.3645570735000, 12.8998246080000, 14.1753736620000, 12.0910467600000, 13.1854277460000, 12.4515449940000, 14.2972183755000, 13.3817490765000, 15.6470456460000, 14.4441143985000, 14.6979759675000, 15.1133204415000, 14.6938200255000, 14.9941104810000, 16.3414740945000, 16.2391122045000, 16.4196783975000, 15.8253425295000, 16.2506765625000, 15.0501356340000, 12.4317025950000, 11.4502026840000, 11.2370425410000, 9.81610206600000, 10.8061799745000, 10.5620890275000, 10.6187718690000, 9.90308998650000, 10.9146927060000, 12.6148996845000, 12.6890887320000, 12.1955006520000, 12.5253720270000, 12.5016806265000, 13.2676470600000, 11.2370425410000, 11.0136340215000, 11.1470456460000, 9.71568188250000, 9.71568188250000, 9.42495184350000, −2.04979731600000, −2.45740824600000, −2.03482098600000, −2.03482098600000, −2.08082421450000, 6.37563000345000, 5.36967539490000, 6.01925583706500, 5.72931590325000, 4.61877186900000, 6.45479408610000, 11.6496192705000] **(8)**

```
> dataplot(VOLL, [VL1N3DMo, VL3r2LDMo, MOML], axes = boxed, view = [-5.7 ..7.0, -2
    ..20], axesfont = [Arial, 18], style = point, axes = boxed);
    #further plot processing in the plot option: linewidth=5; for all plots
```

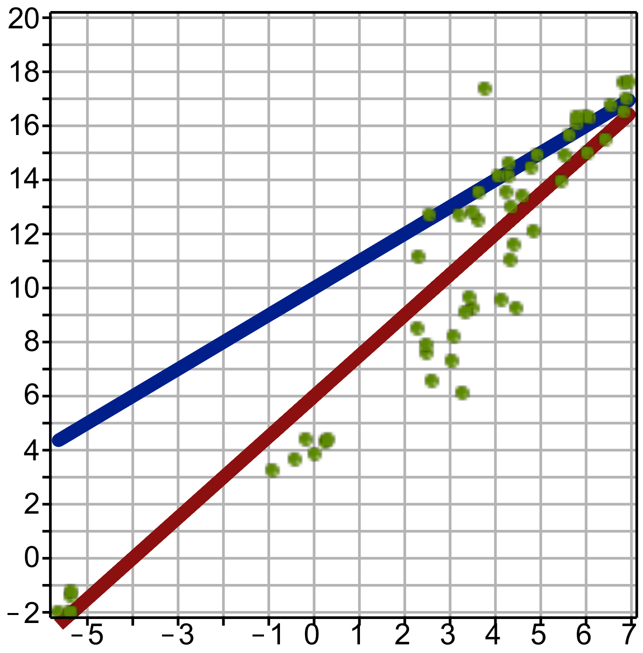

> *DTL1* := -2.0 + ~(*TL*); *dataplot*(*TL*, [*VOLL*, *DTL1*], *axes* = *boxed*, *axesfont* = [*Arial*, 18], *style* = *point*, *axes* = *boxed*);

DTL1 := [2.812913357, 2.462397998, 3.633468456, 4.079181246, 5.322219295, 3.716003344, 5.505149978, 3.934498451, 6.662757832, 5.505149978, 6.079181246, 6.612783857, 5.301029996, 6.544068044, 6.230448921, 6.230448921, 6.690196080, 6.755874856, 6.763427994, 6.755874856, 6.778151250, 6.230448921, 4.230448921, 2.799340549, 3.380211242, 3.301029996, 2.633468456, 1.857332496, 1.732393760, 2.079181246, 2.113943352, 3.146128036, 2.977723605, 2.230448921, 3.113943352, 3.342422681, 4.716003344, 2.204119983, 2.079181246, 1.857332496, 1.477121255, 1.477121255, 1.380211242, 0.409933123, 0.741939078, 0.716003344, 0.113943352, −0.221848750, 1.924279286, 1.556302501, 2.158362492, 2.100370545, 1.732393760, 2.240549248, 5.802773725]

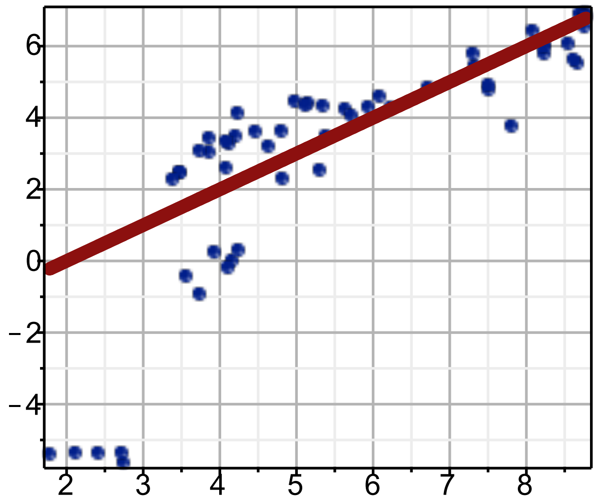

> *dataplot*(*TL*, [*T1LDMo*, *T3r2LDMo*, *MOML*], *axes* = *boxed*, *view* = [1.75 ..8.8, -2 ..20],

axesfont = [*Arial*, 18], *style* = *point*, *axes* = *boxed*);

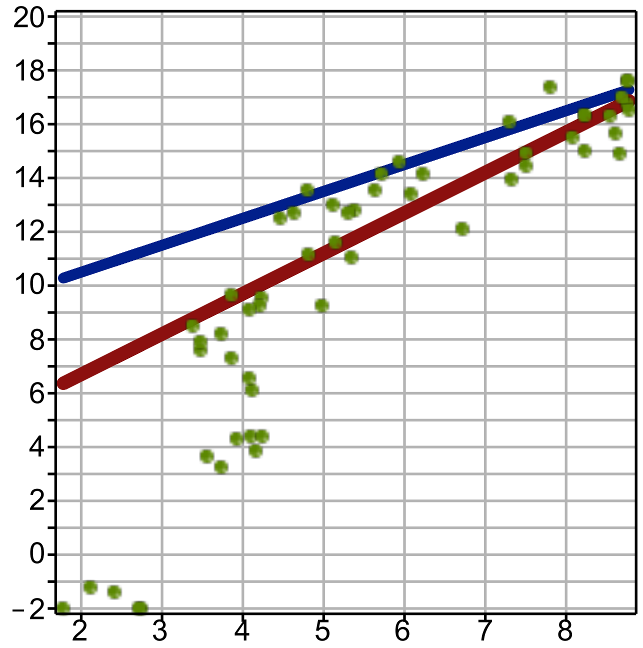

> *dataplot*(*LDVField*, [*MwFld*], *style* = *point*, *view* = [1.5 ..7.5, −3 ..6], *axesfont* = [*Arial*, 18], *axes* = *boxed*);

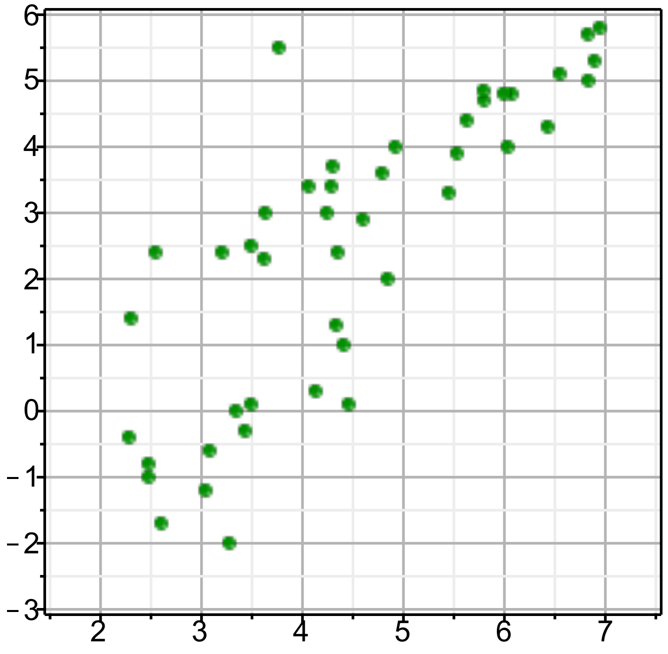

dataplot(*LDTField*, [*MwFld*], *axes* = *boxed*, *style* = *point*, *view* = [3 ..9, −3 ..6], *axesfont* = [*Arial*, 18], *axes* = *boxed*);

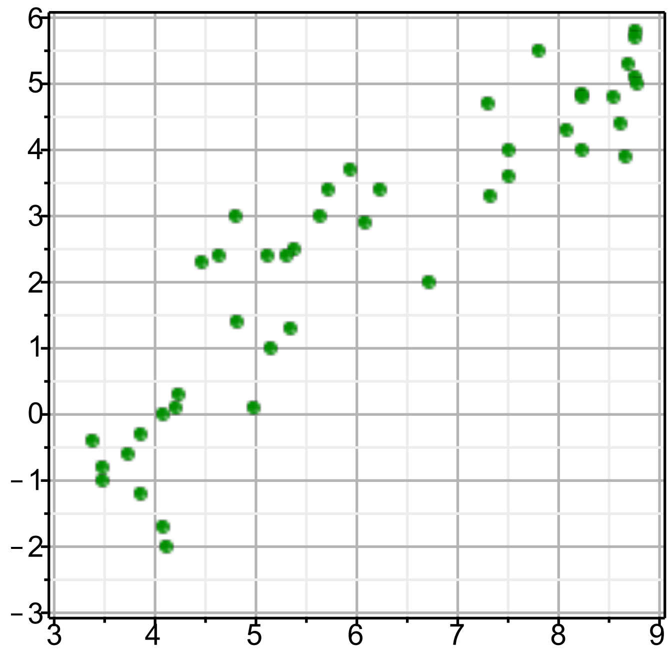

#Linear Regression for DVf-Mw and DT-Mw scalings (shown on the two figures above (Fig. 1d and Fig1.e)

with(*Statistics*) : $\xrightarrow{\text{number of elements}}$

168 **(9)**

LinearFit($a + b \cdot v$, *LDVField*, *MwFld*, *v*, *summarize* = *true*); *# Mmax vs Injected Volume (v)*

```
Summary:
----------------
Model: -3.3377656+1.2997274*v
----------------
Coefficients:
              Estimate  Std. Error  t-value  P(>|t|)
Parameter 1   -3.3378    0.6883     -4.8493   0.0000
Parameter 2    1.2997    0.1472      8.8326   0.0000
----------------
R-squared: 0.6500, Adjusted R-squared: 0.6417
```

$-3.33776560323553 + 1.29972737534090\, v$ **(10)**

LinearFit($a + b \cdot t$, *LDTField*, *MwFld*, *t*, *summarize* = *true*); *# Mmax vs Elapsed Time (t)*

```
Summary:
----------------
Model: -4.2117302+1.0946928*t
----------------
Coefficients:
              Estimate  Std. Error  t-value   P(>|t|)
Parameter 1   -4.2117    0.4940     -8.5255    0.0000
Parameter 2    1.0947    0.0775      14.1337   0.0000
----------------
R-squared: 0.8263, Adjusted R-squared: 0.8221
```

$-4.21173020933126 + 1.09469280032908\, t$ **(11)**

# Please note that the Parameters 2 in both regressions above correspond to the power law exponent 2ξ from the paper. This exponent is significantly better approximated by 1 in the case of DT-Mw scaling (also with significantly smaller standard errors) than for DVf-Mw scaling.

Supplementary Material

Constraining the seismogenic index for the Pohang site.

All our estimates are based on Figure 2 of the main text, corresponding references given in the main text and corresponding data taken directly from the Summary report of the Korean Government Commission on relations between the 2017 Pohang Earthquake and EGS Project; (Geological Society of Korea, Seoul, South Korea, 2019 referred to further as KGC2019). To estimate the seismogenic index at the Pohang geothermal site we use equation (3) of the main text. Strictly speaking, we can apply it in this simple form for a monotonic injection period only. This is the case for the initial part of the mud-loss period in the borehole PX-2. Please note that the mud-loss occurred under a rather enhanced borehole pressure caused by a high mud density ($1.6g/cm^3$; additional pressure of more than $20MPa$). Further, we need moment magnitudes and a well-documented seismicity catalog (including the b value) above the completeness magnitude M_c, which was significantly smaller than Mw 0.9.

The cumulative mud-loss volume (Fig. O-3 of KGC2019; depths 3785 - 3840) was about $650m^3$ (Fig. A-2-1 of KGC2019). It produced at least one event with $M_w \approx 1$ on Nov 30, 2015. The corresponding modification of equation (3) from the main text is

$\Sigma = -\lg V_f(t) + b \approx -2.1$

Here we accepted $b = 0.73$ (KGC2019). Thus, highly probably, at the mud loss domain of PX-2, the seismogenic index was above -2.1. This is a rather high, but not a very high value.

The estimates of the seismogenic index from later injection data will provide values already

altered by the previous injections. Still we attempt to consider them as independent and apply equation (3) of the main text. The next injection in PX-2 (denoted in KGC2019 as the 1st one) was on 29 January-20 February 2016 (Figures 1-2, 6-1, KGC2019). The maximum event was M_w 1.6 on February 07, 2016. The injection depth was $4201 - 4341m$. This event was triggered by the cumulative injected volume of approximately $1300m^3$. The corresponding approximate estimate of the Σ is:

$\Sigma = -\lg V_f(t) + bM_w \approx -3.11 + 0.73 * 1.6 \approx -1.9$

On the other hand, there were 4 events with $M_w \geq 1$ (this corresponds to Fig O-2, KGC2019) caused by the injected volume of 1960 m^3. From here we obtain a close estimate:

$\Sigma \approx -2.0$

The next injection in PX-2 (denoted as the 3rd one) took place more than one year later. It was on March 25th- April 14th of 2017. The total injected volume in this period was approximately 2800 m^3. The approximate bleed-off during this time was $400m^3$. Thus, the net injection volume was approximately 2400 m^3. It induced 10 events with $1 \leq M_w$. This provides an estimate

$\Sigma = 1 - 3.38 + 0.73 = -1.65$

On the other hand, the maximum event induced was M_w3.3 (on April 15th, 2017). This provides us with the following estimate:

$\Sigma = -\lg V_f(t) + bM_w \approx -3.38 + 0.73 * 3.3 \approx -1.0$

The final injection in PX-2 (denoted in KGC2019 as the 5th one) started on August 30th, 2017 and stopped on September 18th, 2017. It was injected about 2300 m^3. The bleed-off volume

was approximately 600 m^3. Thus, we assume the net injection of 1700 m^3. This injection cannot be considered as an independent neither monotonic one. Still we make our estimate. During the injection time and close to it there were 5 events with $1 \leq M_w$ and the maximum one was of $M_w 2$. This yields, respectively:

$\Sigma \approx 0.7 - 3.24 + 0.73 \approx -1.8$

$\Sigma = -\lg V_f(t) + bM_w \approx -3.24 + 0.73 * 2 \approx -1.74$

Let us turn to PX-1. The second official Pohang EGS injection started in this borehole on December 15th and finished on December 28th of 2016. Approximately 4000 m^3 was injected. The bleed off was approximately 1200 m^3. This injection induced maximum $M_w 2.4$ event on 29 December. There were 11 events of $1 \leq M_w$. Thus, we obtain the following quite consistent estimates:

$\Sigma = -\lg V_f(t) + bM_w \approx -3.43 + 0.73 * 2.4 \approx -1.71$

$\Sigma \approx 1.04 - 3.43 + 0.73 \approx -1.66$

Finally, the second injection period in PX-1 (officially called the 4th injection) was from 7th to 14th August of 2017 with the injected volume of about 1750 m^3 and nearly no bleed-off. This injection induced one $M_w 1.2$ event. The seismicity catalog of the second injection in PX-1 is very incomplete. It provides us with one single event only and we do not use it for estimating the seismogenic index.

> **Supplementary Material**

#A MapleWorksheet for computation of Figure 3a,b.

```
> Year := Vector[row](readdata(YearP, float));
Year := [2015., 2015., 2015., 2016., 2016., 2016., 2016., 2016., 2016., 2016., 2016.,
    2016., 2016., 2016., 2016., 2016., 2016., 2016., 2016., 2016., 2016., 2016., 2017., 2017.,
    2017., 2017., 2017., 2017., 2017., 2017., 2017., 2017., 2017., 2017., 2017., 2017., 2017.,
    2017., 2017., 2017., 2017., 2017., 2017., 2017., 2017., 2017., 2017., 2017., 2017.]
```

```
> Vbleed := Vector[row](readdata(Bleed_off_V_Pohang, float));
Vbleed := [0., 0., 0., 0., 0., 0., 0., 195.39, 342.05, 716.69, 815.27, 817.3, 817.3, 817.3, 819.33,
    821.36, 823.39, 823.39, 1163.94, 1238.28, 1244.37, 1331.22, 1331.22, 2740.44, 2936.55,
    2936.55, 2936.55, 2936.55, 2936.55, 3052.73, 3052.73, 3224.69, 3224.69, 3224.69, 3224.69,
    3327.16, 3686.94, 4284.60, 6025.58, 6078.73, 6269.91, 6269.91, 6269.91, 6828.97, 6828.97,
    6828.97, 6828.97, 6828.97, 6828.97, 6828.97]
```

```
> DayOfYear := Vector[row](readdata(Day_of_year_Pohang, float)); Vinj0 :=
      Vector[row](readdata(InjectedV_Pohang, float)); Vinj := 650.0+~( Vinj0);
DayOfYear := [322., 326., 334., 37., 38., 38., 48., 72., 91., 235., 353., 354., 354., 354., 355.,
    356., 357., 357., 359., 360., 363., 364., 364., 98., 105., 105., 105., 105., 105., 106., 106.,
    110., 110., 111., 111., 138., 225., 254., 258., 259., 265., 265., 265., 311., 318., 318., 318.,
    319., 319., 319.]
Vinj0 := [0., 0., 0., 1290.17, 1290.17, 1290.17, 1961.67, 1969.53, 1969.53, 1969.53, 3926.25,
    4807.85, 4807.85, 4807.85, 5111.27, 5621.61, 5650.60, 5650.60, 5650.60, 5650.60, 5876.16,
    5876.16, 5876.16, 7597.64, 8707.68, 8707.68, 8707.68, 8707.68, 8707.68, 8707.68, 8707.68,
    8707.68, 8707.68, 8707.68, 8707.68, 8707.68, 10407.35, 10407.35, 12620.32, 12620.32,
    12798.32, 12798.32, 12798.32, 12798.32, 12798.32, 12798.32, 12798.32, 12798.32, 12798.32,
    12798.32]
Vinj := [650.0, 650.0, 650.0, 1940.17, 1940.17, 1940.17, 2611.67, 2619.53, 2619.53, 2619.53,
    4576.25, 5457.85, 5457.85, 5457.85, 5761.27, 6271.61, 6300.60, 6300.60, 6300.60, 6300.60,
    6526.16, 6526.16, 6526.16, 8247.64, 9357.68, 9357.68, 9357.68, 9357.68, 9357.68, 9357.68,
    9357.68, 9357.68, 9357.68, 9357.68, 9357.68, 9357.68, 11057.35, 11057.35, 13270.32,
    13270.32, 13448.32, 13448.32, 13448.32, 13448.32, 13448.32, 13448.32, 13448.32, 13448.32,
    13448.32, 13448.32]
```

```
> MagnK := Vector[row](readdata(Magnitude_Pohang, float));
MagnK := [0.680, 0.673, 0.972, 1.169, 1.619, 1.286, 1.090, 1.171, 1.586, 1.189, 1.471, 1.407,
    1.104, 0.577, 1.464, 1.345, 1.407, 2.192, 1.056, 1.039, 1.315, 2.355, 0.920, 0.718, 1.558,
    3.294, 2.092, 2.145, 0.869, 1.552, 1.270, 1.210, 0.763, 0.744, 1.262, 1.433, 1.205, 2.028,
    0.816, 1.675, 1.539, 1.273, 1.583, 0.971, 1.665, 1.778, 1.567, 2.197, 2.717, 5.56]
```

```
>  Vcumul := ((Vinj0) − (Vbleed)); Vfluid := ((Vinj) − (Vbleed));
Vcumul := [0., 0., 0., 1290.17000000000, 1290.17000000000, 1290.17000000000,
    1961.67000000000, 1774.14000000000, 1627.48000000000, 1252.84000000000,
    3110.98000000000, 3990.55000000000, 3990.55000000000, 3990.55000000000,
    4291.94000000000, 4800.25000000000, 4827.21000000000, 4827.21000000000,
    4486.66000000000, 4412.32000000000, 4631.79000000000, 4544.94000000000,
    4544.94000000000, 4857.20000000000, 5771.13000000000, 5771.13000000000,
    5771.13000000000, 5771.13000000000, 5771.13000000000, 5654.95000000000,
    5654.95000000000, 5482.99000000000, 5482.99000000000, 5482.99000000000,
    5482.99000000000, 5380.52000000000, 6720.41000000000, 6122.75000000000,
```

6594.74000000000, 6541.59000000000, 6528.41000000000, 6528.41000000000,
6528.41000000000, 5969.35000000000, 5969.35000000000, 5969.35000000000,
5969.35000000000, 5969.35000000000, 5969.35000000000, 5969.35000000000]

Vfluid := [650., 650., 650., 1940.17000000000, 1940.17000000000, 1940.17000000000,
2611.67000000000, 2424.14000000000, 2277.48000000000, 1902.84000000000,
3760.98000000000, 4640.55000000000, 4640.55000000000, 4640.55000000000,
4941.94000000000, 5450.25000000000, 5477.21000000000, 5477.21000000000,
5136.66000000000, 5062.32000000000, 5281.79000000000, 5194.94000000000,
5194.94000000000, 5507.20000000000, 6421.13000000000, 6421.13000000000,
6421.13000000000, 6421.13000000000, 6421.13000000000, 6304.95000000000,
6304.95000000000, 6132.99000000000, 6132.99000000000, 6132.99000000000,
6132.99000000000, 6030.52000000000, 7370.41000000000, 6772.75000000000,
7244.74000000000, 7191.59000000000, 7178.41000000000, 7178.41000000000,
7178.41000000000, 6619.35000000000, 6619.35000000000, 6619.35000000000,
6619.35000000000, 6619.35000000000, 6619.35000000000, 6619.35000000000]

$$> TimeK1 := 0.+\sim(Year) + \frac{(DayOfYear)}{365.};$$

TimeK1 := [2015.88219178069, 2015.89315068480, 2015.91506849302, 2016.10136986300,
2016.10410958903, 2016.10410958903, 2016.13150684930, 2016.19726027394,
2016.24931506846, 2016.64383561635, 2016.96712328753, 2016.96986301356,
2016.96986301356, 2016.96986301356, 2016.97260273958, 2016.97534246561,
2016.97808219164, 2016.97808219164, 2016.98356164369, 2016.98630136972,
2016.99452054780, 2016.99726027383, 2016.99726027383, 2017.26849315065,
2017.28767123284, 2017.28767123284, 2017.28767123284, 2017.28767123284,
2017.28767123284, 2017.29041095886, 2017.29041095886, 2017.30136986297,
2017.30136986297, 2017.30410958900, 2017.30410958900, 2017.37808219173,
2017.61643835607, 2017.69589041086, 2017.70684931497, 2017.70958904099,
2017.72602739715, 2017.72602739715, 2017.72602739715, 2017.85205479440,
2017.87123287659, 2017.87123287659, 2017.87123287659, 2017.87397260261,
2017.87397260261, 2017.87397260261]

> *N09* := *Vector*[*row*](*readdata*(*Mnumber_Pohang*, *float*)); *#number of events with Mw>=0.9;*

N09 := [0., 0., 1., 2., 3., 4., 5., 6., 7., 8., 9., 10., 11., 11., 12., 13., 14., 15., 16., 17., 18., 19.,
20., 20., 21., 22., 23., 24., 24., 25., 26., 27., 27., 27., 28., 29., 30., 31., 31., 32., 33., 34.,
35., 36., 37., 38., 39., 40., 41., 42.]

> *MagnMaxObs* := *Vector*[*row*](*readdata*(*Pohang_Mmax_obs_time*, *float*)); *SImaxObs* :=
Vector[*row*](*readdata*(*Pohang_Simax_obs_time*, *float*));
#Mmax observed during the ellapsed time after the start of injections and mudloss; sup(SI)

MagnMaxObs := [0.680, 0.680, 0.972, 1.619, 1.619, 1.619, 1.619, 1.619, 1.619, 1.619, 1.619,
1.619, 1.619, 1.619, 1.619, 1.619, 1.619, 2.192, 2.192, 2.192, 2.192, 2.192, 2.192, 2.192,
2.192, 3.294, 3.294, 3.294, 3.294, 3.294, 3.294, 3.294, 3.294, 3.294, 3.294, 3.294, 3.294,
3.294, 3.294, 3.294, 3.294, 3.294, 3.294, 3.294, 3.294, 3.294, 3.294, 3.294, 3.294, 3.294]

SImaxObs := [−2.1, −2.1, −2.1, −2.1, −1.9, −1.9, −1.9, −1.9, −1.9, −1.9, −1.66,
−1.66, −1.66, −1.66, −1.66, −1.66, −1.66, −1.66, −1.66, −1.66, −1.66, −1.66,
−1.66, −1.65, −1.65, −1.0, −1.0, −1.0, −1.0, −1.0, −1.0, −1.0, −1.0, −1.0, −1.0,
−1.0, −1.0, −1.0, −1.0, −1.0, −1.0, −1.0, −1.0, −1.0, −1.0, −1.0, −1.0, −1.0,
−1.0, −1.0]

$$SI09 := 0.73 \cdot 0.9 +\sim((\log10\sim(N09)) - (\log10\sim(Vfluid)));$$

Seismogenic Index computed in the approximation of a continuous single injection of a monotonic rate.

$SI09 := [\text{Float}(-\infty), \text{Float}(-\infty), -2.15591335664286, -2.32980978929584, -2.15371853029584, -2.02877979369584, -2.06094829608514, -1.94940644738456, -1.85535653184579, -1.71931228523950, -1.96405851455345, -2.00956945637589, -1.96817677137589, -1.96817677137589, -1.95771622233698, -1.96547307158496, -1.93543135639725, -1.90546813339725, -1.84956083734559, -1.81690067336662, -1.81050862499203, -1.77982693495106, -1.75755053995106, -1.78290085264747, -1.82839216758292, -1.80818878158292, -1.78888362658292, -1.77040022058292, -1.77040022058292, -1.74474163784721, -1.72770829884721, -1.69930849255960, -1.69930849255960, -1.69930849255960, -1.68351422555960, -1.66095676412253, -1.73337039239568, -1.68240335095166, -1.71166111008028, -1.69467494185237, -1.68051431974429, -1.66754934274429, -1.65496021574429, -1.60751284413866, -1.59561362113866, -1.58403174813866, -1.57275073813866, -1.56175535413866, -1.55103148813866, -1.54056605513866]$

$SImaxCont := Vector[row](readdata(SImax\_contin\_Pohang, float));$

This array is obtained from SI09 by taking its time-dependent (number of entry - dependent) supremum

$SImaxCont := [-2.155913357, -2.155913357, -2.155913357, -2.153718530, -2.153718530, -2.028779794, -2.028779794, -1.949406447, -1.855356532, -1.719312285, -1.699308493, -1.699308493, -1.699308493, -1.683514226, -1.660956764, -1.660956764, -1.660956764, -1.660956764, -1.660956764, -1.660956764, -1.660956764, -1.654960216, -1.607512844, -1.595613621, -1.584031748, -1.572750738, -1.561755354, -1.551031488, -1.540566055]$

$Mmax := 0.9 +\sim\left(\frac{(\log10\sim(N09))}{0.73}\right); SIcum := 0.73 \cdot\sim(MagnMaxObs) - (\log10\sim(Vfluid));$

$Mmax := [\text{Float}(-\infty), \text{Float}(-\infty), 0.900000000000000, 1.31236985721401, 1.55359076000680, 1.72473971429103, 1.85749315678599, 1.96596061722081, 2.05766854819989, 2.13710957150504, 2.20718152001361, 2.26986301400000, 2.32656532223165, 2.32656532223165, 2.37833047429784, 2.42594979759598, 2.47003840582486, 2.51108391679279, 2.54947942913001, 2.58554646749411, 2.61955137709063, 2.65171726202921, 2.68223287162497, 2.68223287162497, 2.71125930861766, 2.73893517985662, 2.76538059767066, 2.79070033192280, 2.79070033192280, 2.81498631411993, 2.83831965522095, 2.86077227988343, 2.86077227988343, 2.86077227988343, 2.88240826207997, 2.90328492920785, 2.92345377441776, 2.94296122510699, 2.94296122510699, 2.96184928538511, 2.98015608264942, 2.99791632511908, 3.01516170457492, 3.03192123471560, 3.04822154019864, 3.06408711965418, 3.07954055801375, 3.09460272788007, 3.10929295528056, 3.12362916487276]$

$SIcum := [-2.31651335664286, -2.31651335664286, -2.10335335664286, -2.10596978499584, -2.10596978499584, -2.10596978499584, -2.23504830038514, -2.20268769778456, -2.17558457184579, -2.09753227223950, -2.39343102395345, -2.48469945637589, -2.48469945637589, -2.48469945637589, -2.51202746833698,$

−2.55454642358496, −2.55668939239725, −2.13839939239725, −2.11052082034559, −2.10418959436662, −2.12262112999203, −2.11542053595106, −2.11542053595106, −2.14077084864747, −2.20745146258292, −1.40299146258292, −1.40299146258292, −1.40299146258292, −1.40299146258292, −1.39506164684721, −1.39506164684721, −1.38305225655960, −1.38305225655960, −1.38305225655960, −1.38305225655960, −1.37573476212253, −1.46287164739568, −1.42614504495166, −1.45540280408028, −1.45220491985237, −1.45140825974429, −1.45140825974429, −1.45140825974429, −1.41619534513866, −1.41619534513866, −1.41619534513866, −1.41619534513866, −1.41619534513866, −1.41619534513866, −1.41619534513866]

dataplot(*TimeK1*, [*SImaxCont*, *SImaxObs*, *MagnK*, *Mmax*], *style* = *point*, *axesfont* = [*Arial*, 18], *axes* = *boxed*);

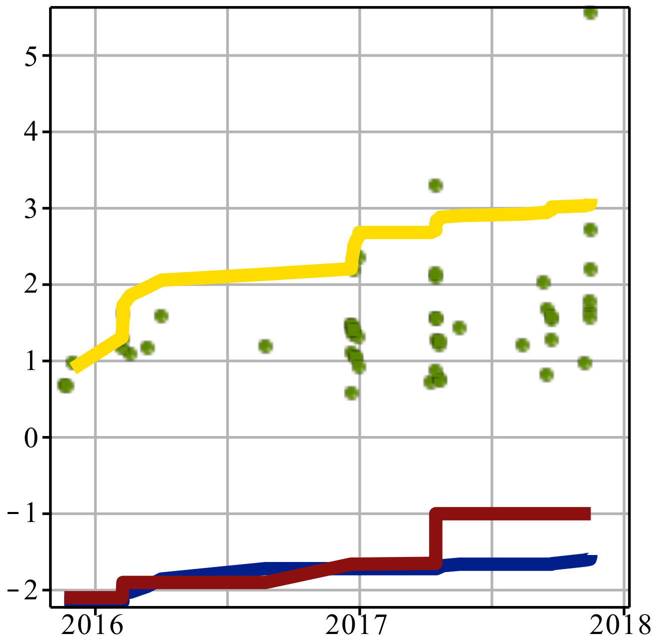

Wcont73ge5 := 100·~(1.+~(−1.· exp~((−1)·(*Vfluid*)·~(exp~(ln(10.)·(−0.73·5.5 +~(*SImaxCont*))))))); *WObs73ge5max* := 100·~(1.+~(−1.· exp~((−1)·(*Vfluid*)·~(exp ~(ln(10.)·(−0.73·5.5+~(*SImaxObs*)))))));

Wcont73ge5 := [0.0438434556865985, 0.0438434556865985, 0.0438434556865985, 0.131472708221003, 0.131472708221003, 0.175258521547739, 0.235844564987464, 0.262772565787639, 0.306500812576116, 0.350209888069009, 0.691009301018497, 0.851923586525694, 0.851923586525694, 0.851923586525694, 0.907001843730659, 0.999824897102042, 1.00474566376909, 1.00474566376909, 0.942570158624578, 0.928992431078202, 0.969071866590032, 0.953213328189462, 0.953213328189462, 1.01021918176122, 1.17687679915297, 1.17687679915297, 1.17687679915297, 1.17687679915297, 1.17687679915297, 1.15570663741281, 1.15570663741281, 1.17705079688372, 1.17705079688372, 1.17705079688372, 1.22037819229011, 1.26368659487182, 1.54228322585119, 1.41811201933522, 1.51618673211320, 1.50514758045066, 1.50240992861375, 1.50240992861375, 1.52313853597816, 1.56631419856871, 1.60947092695467, 1.65260873671030, 1.69572763011924, 1.73882761906415, 1.78190871512423, 1.82497091770654]

WObs73ge5max := [0.0498660595524059, 0.0498660595524059, 0.0498660595524059, 0.148770401036336, 0.235682590712072, 0.235682590712072, 0.317123746254777,

0.294386344253228, 0.276600682705552, 0.231153165596409, 0.791728191648111, 0.975981242119250, 0.975981242119250, 0.975981242119250, 1.03903791820906, 1.14529534644109, 1.15092789387583, 1.15092789387583, 1.07975579353280, 1.06421254273874, 1.11009301283947, 1.09193945929226, 1.09193945929226, 1.18398739380814, 1.37911158775605, 6.01466089067118, 6.01466089067118, 6.01466089067118, 6.01466089067118, 5.90911648356645, 5.90911648356645, 5.75268082222038, 5.75268082222038, 5.75268082222038, 5.75268082222038, 5.65933804540166, 6.87261609051841, 6.33337132287463, 6.75948742361666, 6.71160022612612, 6.69972147721744, 6.69972147721744, 6.69972147721744, 6.19446177950963, 6.19446177950963, 6.19446177950963, 6.19446177950963, 6.19446177950963, 6.19446177950963, 6.19446177950963]

Wcont65ge5 := 100·~(1.+~(−1.· exp~((−1)·(*Vfluid*)·~(exp~(ln(10.)·(−0.65·5.5 +~(*SImaxCont*))))))); *WObs65ge5* := 100·~(1.+~(−1.· exp~((−1)·(*Vfluid*)·~(exp ~(ln(10.)·(−0.65·5.5+~(*SImaxObs*)))))));

Wcont65ge5 := [0.120708472051745, 0.120708472051745, 0.120708472051745, 0.361688476503674, 0.361688476503674, 0.481960359424061, 0.648226949805020, 0.722068767674522, 0.841905640660190, 0.961597862246633, 1.89168252919324, 2.32889691612382, 2.32889691612382, 2.32889691612382, 2.47826243691618, 2.72965789446415, 2.74297342021339, 2.74297342021339, 2.57464201349358, 2.53785756437893, 2.64641431967169, 2.60347004624931, 2.60347004624931, 2.75778331933101, 3.20802752591361, 3.20802752591361, 3.20802752591361, 3.20802752591361, 3.20802752591361, 3.15090774397550, 3.15090774397550, 3.20849690493875, 3.20849690493875, 3.20849690493875, 3.32533244795682, 3.44202696974230, 4.19056125297604, 3.85739597542190, 4.12060256488173, 4.09099935273700, 4.08365701030530, 4.08365701030530, 4.13924192767048, 4.25495399334102, 4.37052637268610, 4.48595925379155, 4.60125278896595, 4.71640715601054, 4.83142253169389, 4.94629906010556]

WObs65ge5 := [0.137282468871525, 0.137282468871525, 0.137282468871525, 0.409213234471928, 0.647782679019304, 0.647782679019304, 0.871003779477253, 0.808715279462091, 0.759974566669497, 0.635358642854655, 2.16548778853892, 2.66512085345291, 2.66512085345291, 2.66512085345291, 2.83573554481494, 3.12280888659806, 3.13801111833764, 3.13801111833764, 2.94580606560120, 2.90379817885158, 3.02776344584242, 2.97872609277359, 2.97872609277359, 3.22720799737521, 3.75260138822691, 15.7050826349615, 15.7050826349615, 15.7050826349615, 15.7050826349615, 15.4441044619875, 15.4441044619875, 15.0563424675022, 15.0563424675022, 15.0563424675022, 15.0563424675022, 14.8244322582467, 17.8075178283736, 16.4900380521297, 17.5322281071826, 17.4155217132114, 17.3865556154575, 17.3865556154575, 17.3865556154575, 16.1484912333059, 16.1484912333059, 16.1484912333059, 16.1484912333059, 16.1484912333059, 16.1484912333059, 16.1484912333059]

dataplot(*TimeK1*, [*Wcont73ge5*, *WObs73ge5max*, *MagnK*, *Wcont65ge5*, *WObs65ge5*], *style* = *point*, *axesfont* = [*Arial*, 18], *axes* = *boxed*);

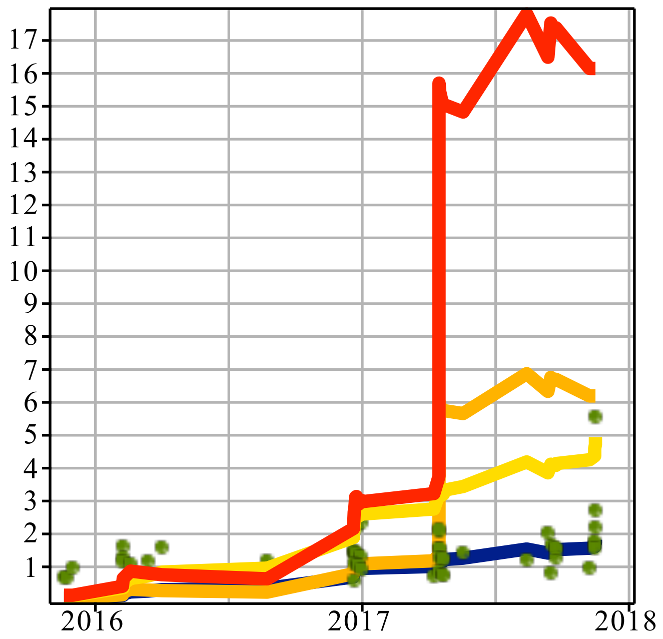
17
16
15
14
13
12
11
10
9
8
7
6
5
4
3
2
1
2016
2017
2018

> **Supplementary Material**

#A MapleWorksheet for computation of Figure 3c,d.

$Wr(b, SI, Qc, Mw) := 100\cdot\left(1 - \exp\left(-Qc\cdot 10^{SI - b\cdot Mw}\right)\right);$

$$Wr := (b, SI, Qc, Mw) \rightarrow 100 - 100\,\mathrm{e}^{-Qc\,10^{SI - b\,Mw}}$$

plot([*Wr*(0.65, -1.0, 6441, *Mw*), *Wr*(0.65, -2.0, 6441, *Mw*), *Wr*(1.44, 0.25, 11570, *Mw*)], *Mw* = 0 ..7, *axes* = *boxed*, *axesfont* = [*Arial*, 18]);

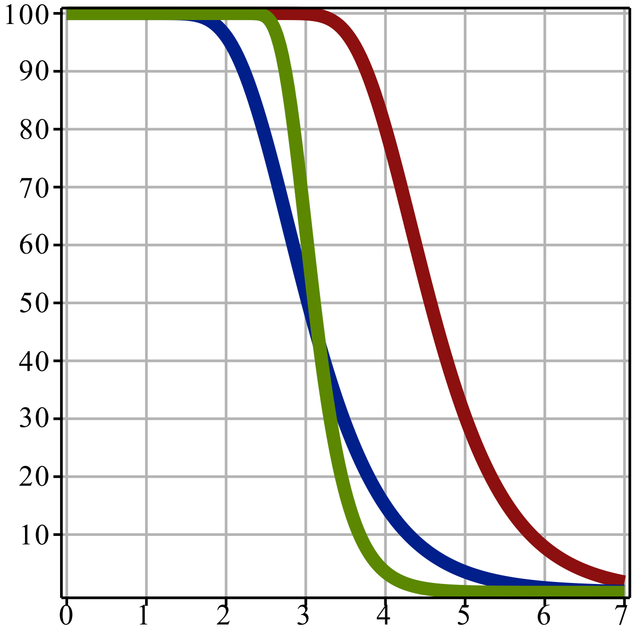

$Wrst(Mw, SI, b, Qc, \tau, pd) := 100\cdot\left(1 - \exp\left(-Qc\cdot\left(\frac{1 - (\tau)^{1 - pd}}{pd - 1}\right)\cdot 10^{SI - b\cdot Mw}\right)\right);$

$$Wrst := (Mw, SI, b, Qc, \tau, pd) \rightarrow 100 - 100\,\mathrm{e}^{-\frac{Qc\,(1 - \tau^{1 - pd})\,10^{SI - b\,Mw}}{pd - 1}}$$

$Wrtl(Mw, M0, b, \tau, pd) := 100\cdot\left(1 - \exp\left(-\left(\frac{1 - (\tau)^{1 - pd}}{pd - 1}\right)\cdot 10^{b\cdot M0 - b\cdot Mw}\right)\right);$

$$Wrtl := (Mw, M0, b, \tau, pd) \rightarrow 100 - 100\,\mathrm{e}^{-\frac{(1 - \tau^{1 - pd})\,10^{M0\,b - b\,Mw}}{pd - 1}}$$

plot([*Wrst*(*Mw*, -1, 0.65, 6441, 10, 2), *Wrtl*(*Mw*, 3.3, 0.65, 10, 2), *Wrtl*(*Mw*, 2.3, 0.65, 10, 2)], *Mw* = 0 ..7, *style* = *line*, *axes* = *boxed*, *axesfont* = [*Arial*, 18]);

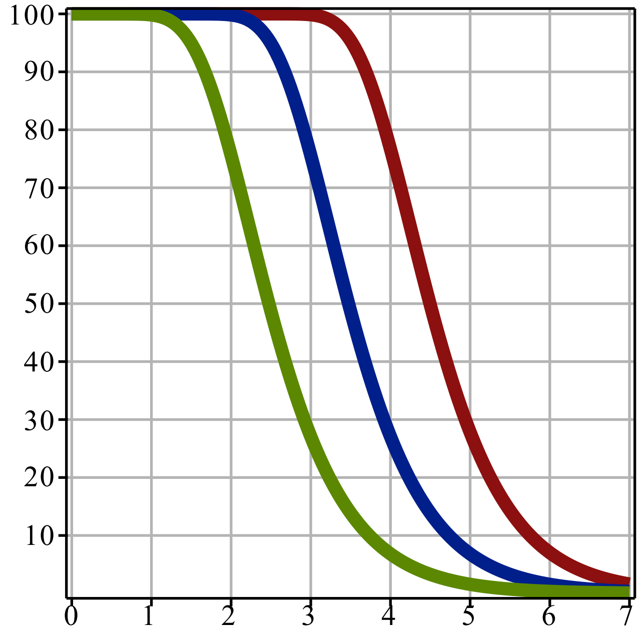

100
90
80
70
60
50
40
30
20
10
0
1
2
3
4
5
6
7

```
> Supplementary Material


    #A MapleWorksheet for computation of Figure 4a,b,c. All relevant
    data are included in this file.

> MagnDen := Vector[row](readdata(magnitude_denver, float))                ;
    #Two artificial vanishingly small events  with Mw -5.0, year 1963, Day 273 and year 1964, Day
    259 (approximate start and stop of the injection break) added to the vector (entries #12 and 13)
    for convenient plotting

> #Years of events
> Year := Vector[row](readdata(year_denver, float));
[1962., 1962., 1962., 1962., 1963., 1963., 1963., 1963., 1963., 1963., 1963., 1963., 1964., 1965.,
    1965., 1965., 1965., 1965., 1965., 1965.]
> #Days of events
> DayOfYear := Vector[row](readdata(day_of_year_denver, float));
[169., 219., 338., 339., 30., 98., 114., 145., 156., 183., 209., 273., 259., 5., 47., 47., 212., 257.,
    273., 273.]
> #Cumulative Injected Volume (m**3)
> Vinj := Vector[row](readdata(injectedV_denver, float)); Vfluid := Vinj; Vfluid_plot :=
    (Vinj)/100000.;
[1.1 10^5, 1.3 10^5, 2.4 10^5, 2.4 10^5, 2.8 10^5, 3.6 10^5, 3.7 10^5, 3.9 10^5, 3.9 10^5, 4.0 10^5, 4.3 10^5,
    4.3 10^5, 4.3 10^5, 4.5 10^5, 4.7 10^5, 4.7 10^5, 5.9 10^5, 6.2 10^5, 6.4 10^5, 6.5 10^5]
[1.1 10^5, 1.3 10^5, 2.4 10^5, 2.4 10^5, 2.8 10^5, 3.6 10^5, 3.7 10^5, 3.9 10^5, 3.9 10^5, 4.0 10^5, 4.3 10^5,
    4.3 10^5, 4.3 10^5, 4.5 10^5, 4.7 10^5, 4.7 10^5, 5.9 10^5, 6.2 10^5, 6.4 10^5, 6.5 10^5]
[1.1, 1.3, 2.4000000000000004, 2.4000000000000004, 2.8000000000000003, 3.6, 3.7,
    3.9000000000000004, 3.9000000000000004, 4.0, 4.300000000000001, 4.300000000000001,
    4.300000000000001, 4.5, 4.7, 4.7, 5.9, 6.2, 6.4, 6.500000000000001]
> TimeDenver := 0. +~(Year) + (DayOfYear)/365.;
[1962.463013698563, 1962.599999999913, 1962.926027397126, 1962.928767123153,
    1963.08219178081, 1963.268493150646, 1963.312328767078, 1963.397260273915,
    1963.427397260212, 1963.501369862941, 1963.572602739643, 1963.747945205371,
    1964.709589040993, 1965.013698630135, 1965.128767123269, 1965.128767123269,
    1965.580821917724, 1965.704109588939, 1965.747945205371, 1965.747945205371]

>
dataplot(TimeDenver, [Vfluid_plot, MagnDen], style = point, axes = boxed, axesfont = [Arial, 18]);
```

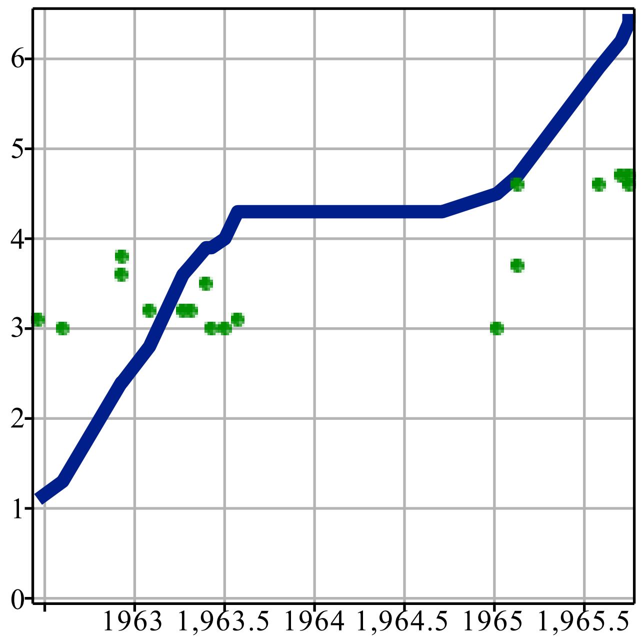

N3 := *Vector*[*row*](*readdata*(*Mnumber_denver*, *float*)); *Mmax* := $3.0 +\sim\left(\frac{(\log 10\sim(N3))}{0.85}\right)$;

# Number of events with Mw >= 3 and the expected (most probable) Mmax

[1. 2. 3. 4. 5. 6. 7. 8. 9. 10. 11. 11. 11. 12. 13. 14. 15. 16. 17. 18.]

[3.0, 3.3541529360468165, 3.561319123064207, 3.708305871975986, 3.8223176519531834, 3.915472059111023, 3.9942329880364476, 4.062458808022802, 4.122638246128414, 4.176470588, 4.225167864460849, 4.225167864460849, 4.225167864460849, 4.269624995040193, 4.310521590326131, 4.348385924436205, 4.38363677501739, 4.41661174442256, 4.447586965592835, 4.476791182057583]

\>

# SImax_ind: supremum of the Seismogenic Index computed using eq. 3 (see Methods); The first and the second injection periods are considered as independent. SImax: supremum # of the Seismogenic Index computed using eq. 3 (see Methods); The first and the second injection periods are considered as parts of a continuous injection.

SImax_ind := *Vector*[*row*](*readdata*(*denver_SImax_65independent*, *float*));

[-2.4, -2.4, -2.34, -2.17, -2.17, -2.17, -2.17, -2.17, -2.17, -2.17, -2.17, -2.17, -2.17, -2.17, -0.7, -0.7, -0.7, -0.7, -0.7, -0.7]

SImax := *Vector*[*row*](*readdata*(*denver_SImax*, *float*));

[-2.4, -2.4, -2.34, -2.17, -2.17, -2.17, -2.17, -2.17, -2.17, -2.17, -2.17, -2.17, -2.17, -2.17, -1.76, -1.76, -1.76, -1.76, -1.76, -1.76]

dataplot(*TimeDenver*, [*SImax*, *SImax_ind*, *MagnDen*, *Mmax*], *style* = *point*, *axes* = *boxed*, *axesfont* = [*Arial*, 18]);

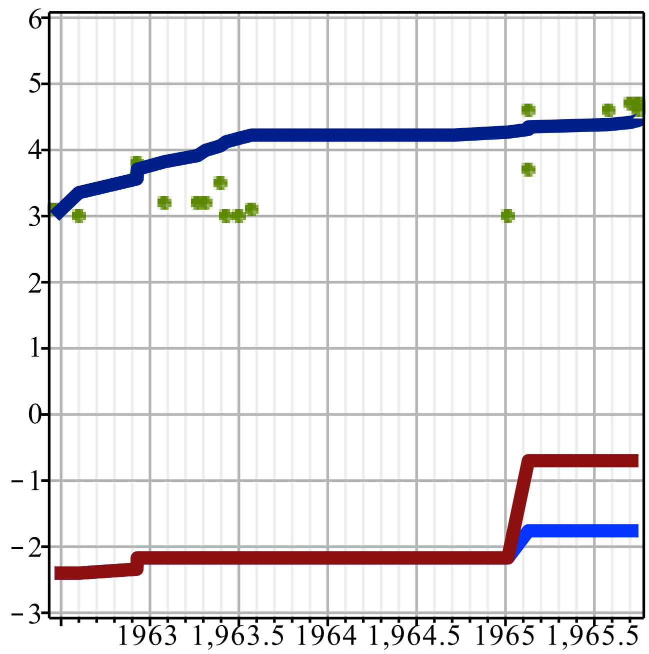

MagnDen_plot ≔ (*MagnDen*)·10;

[31.0, 30.0, 36.0, 38.0, 32.0, 32.0, 32.0, 35.0, 30.0, 30.0, 31.0, −50.0, −50.0, 30.0, 46.0, 37.0, 46.0, 47.0, 47.0, 46.0]

Wge5max ≔ 100·~(1.+~(−1.· exp~((−1)·(*Vfluid*)·~(exp~(ln(10.)·(−0.85·5.5 +~(*SImax*))))))); *#Exceedance Probability accounting for SImax*

[0.9212647670294505, 1.0878532938641605, 2.2918509091496064, 3.371209899167249, 3.921923754100698, 5.013953346276045, 5.149581412338355, 5.420256841789717, 5.420256841789717, 5.555304757827139, 5.9592926187969635, 5.9592926187969635, 5.9592926187969635, 6.227657368635054, 15.85449482593444, 15.85449482593444, 19.482574349847404, 20.36488333589309, 20.947711467567963, 21.237523990635687]

Wge5max_ind ≔ 100·~(1.+~(−1.· exp~((−1)·(*Vfluid*)·~(exp~(ln(10.)·(−0.85·5.5 +~(*SImax_ind*))))))); *#Exceedance Probability accounting for SImax_Ind*

[0.9212647670294505, 1.0878532938641605, 2.2918509091496064, 3.371209899167249, 3.921923754100698, 5.013953346276045, 5.149581412338355, 5.420256841789717, 5.420256841789717, 5.555304757827139, 5.9592926187969635, 5.9592926187969635, 5.9592926187969635, 6.227657368635054, 86.2202982738058, 86.2202982738058, 91.69248259915486, 92.67969594400671, 93.27176692821418, 93.5495950370516]

dataplot(*TimeDenver*, [*Wge5max*, *Wge5max_ind*, *MagnDen_plot*], *style* = *point*, *axes* = *boxed*, *axesfont* = [*Arial*, 18]);

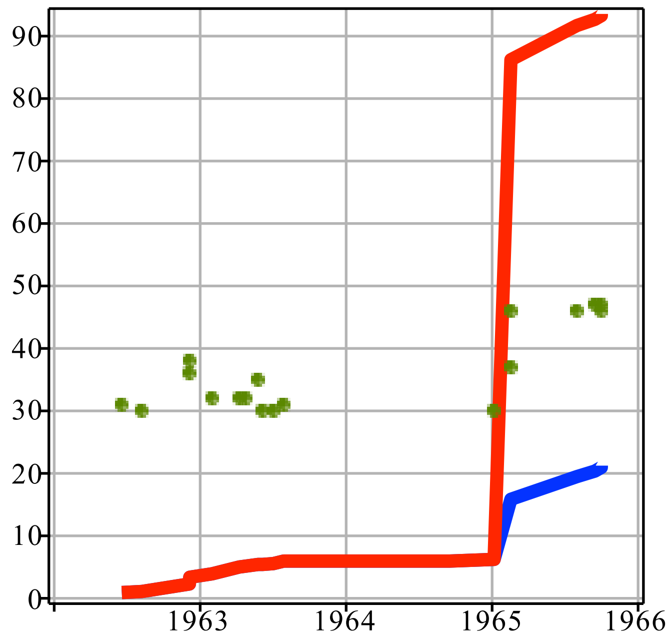

90
80
70
60
50
40
30
20
10
0
1963
1964
1965
1966
