# Supplementary material for: Magnitude and nucleation time of the 2017 Pohang Earthquake point to its predictable artificial triggering
Source: Nat Commun. 2021 Nov 4;12:6397. doi: 10.1038/s41467-021-26679-w (PMC8568929; doi:10.1038/s41467-021-26679-w)
Supplement: Supplementary file 3 — Description of Additional Supplementary Files [file 41467_2021_26679_MOESM3_ESM.pdf]

Description of Additional Supplementary Files

File Name: Supplementary Data 1
Description: Magnitudes, injection volumes and elapsed times until occurrences of maximum induced earthquakes.

File Name: Supplementary Data 2
Description: Induced seismicity, hydraulic data and Seismogenic Index of the Pohang EGS.
